# Pass/Fail Grading and United States Medical Licensing Examination (USMLE) Step 1 Reform: Implications for Clinical-Year Assessment and USMLE Step 2 Clinical Knowledge (CK) Performance

**DOI:** 10.7759/cureus.103358

**Published:** 2026-02-10

**Authors:** Mani Khorsand Askari, Kevin Wunderly, Hoda Shabpiray

**Affiliations:** 1 Internal Medicine, The University of Toledo College of Medicine and Life Sciences, Toledo, USA

**Keywords:** clinical year assessment, medical student well being, pass/fail grading, programmatic assessment, residency program selection, usmle step 1 examination, usmle step 2 ck, workplace-based assessment

## Abstract

The transition to pass/fail grading in undergraduate medical education (UME), alongside the change to pass/fail reporting for the United States Medical Licensing Examination (USMLE) Step 1, represents a substantial shift in assessment across the medical education continuum. These reforms were motivated by concerns regarding learner well-being, excessive competition, and misalignment between assessment practices and educational goals. This narrative review synthesizes empirical, survey-based, and conceptual literature to examine the effects of preclinical pass/fail grading and Step 1 pass/fail reporting on learner outcomes, clinical-year assessment, and USMLE Step 2 Clinical Knowledge (CK) performance. Across national and institutional studies, preclinical pass/fail grading is associated with improved learner well-being without evidence of inferior Step 1 or Step 2 CK performance. Early post-reform evidence suggests that Step 1 pass/fail reporting has redistributed evaluative emphasis toward Step 2 CK and clinical-year performance measures, raising concerns regarding variability and equity in workplace-based assessment. Collectively, the literature underscores the importance of transparent, equitable, and programmatic assessment systems during the clinical years.

## Introduction and background

Preclinical grading systems function as powerful policy levers in undergraduate medical education (UME), shaping feedback, motivation, and progression through medical school [1-3]. Over the past two decades, many U.S. medical schools have adopted pass/fail grading during the preclinical years, while others retain tiered or hybrid systems [1]. In parallel, the United States Medical Licensing Examination (USMLE) Step 1 transitioned from numeric scoring to pass/fail reporting in January 2022, intensifying scrutiny of how assessment signals function across the continuum of medical education [4,5].

Concerns surrounding a score-centric culture tied to Step 1, including adverse effects on learner well-being, distorted learning behaviors, and equity implications, were central to the rationale for this reform [3-5]. However, whether these changes meaningfully reduce assessment-related pressure or instead redistribute it to downstream assessments during the clinical years and residency selection process remains uncertain [5-9]. Emerging literature suggests that removal of a highly standardized numeric metric may shift evaluative emphasis toward less standardized domains, particularly clinical-year performance and USMLE Step 2 Clinical Knowledge (CK).

While prior studies have examined preclinical pass/fail grading, Step 1 score reporting, or residency selection in isolation, fewer analyses have integrated these reforms across the UME continuum. This narrative review uniquely synthesizes evidence linking preclinical pass/fail grading and USMLE Step 1 pass/fail reporting to downstream reliance on USMLE Step 2 CK and clinical-year assessment, with particular attention to variability and equity in workplace-based evaluation. By integrating evidence across grading reforms, licensing examination reporting changes, and clinical-year assessment practices, this review provides a practical framework for clinical-year faculty and academic leaders navigating assessment design in the post-Step 1 numeric era.

## Review

Methods

Literature Search Strategy

This narrative review synthesized empirical, survey-based, and conceptual literature examining preclinical pass/fail grading, the transition to USMLE Step 1 pass/fail score reporting, determinants of USMLE Step 2 CK performance, and clinical-year assessment practices relevant to residency selection. Targeted literature searches were conducted in PubMed/MEDLINE and Google Scholar, with the most recent search update completed in December 2025. Searches were restricted to English-language publications, with no restriction on start date.

Four structured domains guided the literature identification process: preclinical pass/fail grading systems, USMLE Step 1 pass/fail reporting and residency selection implications, predictors and correlates of USMLE Step 2 CK performance, and variability, reliability, and equity in clinical-year assessment.

Example PubMed search terms included combinations of: “pass/fail grading,” “USMLE Step 1,” “USMLE Step 2 CK,” “clinical clerkship assessment,” “NBME subject examination,” “clinical learning environment,” and “residency selection.” Reference lists of key articles were manually reviewed to identify additional relevant studies not captured through the initial database searches.

Study Selection and Inclusion Criteria

Titles and abstracts were screened for relevance to undergraduate medical education and residency selection. Full-text review was performed when articles addressed learner outcomes, assessment practices, grading systems, or implications for residency selection. Articles were included if they met one or more of the following criteria: evaluated outcomes associated with preclinical pass/fail grading or grading reform, examined perceived or measured effects of USMLE Step 1 pass/fail reporting, analyzed predictors or correlates of USMLE Step 2 CK performance, or assessed clinical-year evaluation practices, grading variability, reliability, or equity-related outcomes.

Priority was given to studies using national datasets, multi-institutional or longitudinal designs, validated survey instruments, or widely cited conceptual frameworks in medical education. Editorials, perspective pieces, and conceptual articles were included when they provided influential theoretical context or addressed system-level implications relevant to assessment reform. When full-text articles were unavailable, abstracts were used solely to inform high-level thematic trends and were not relied upon for detailed methodological or quantitative conclusions.

Risk of Bias and Methodological Considerations

Consistent with the narrative scope of this review, formal risk-of-bias assessment tools (e.g., Cochrane Risk of Bias or ROBINS-I) were not applied. Instead, methodological limitations were considered qualitatively during synthesis. Particular attention was paid to study design (e.g., cross-sectional surveys versus longitudinal analyses), reliance on self-reported outcomes, single-institution versus multi-institution sampling, and potential sources of selection or response bias. Findings were interpreted with these limitations in mind, and conclusions were framed cautiously when evidence was observational or context-dependent.

Narrative Synthesis Approach

Given substantial heterogeneity across study designs, institutional contexts, and outcome measures, results were synthesized using a qualitative narrative approach rather than quantitative pooling or meta-analysis. Evidence was organized thematically according to the four predefined domains, with emphasis placed on areas of convergence across studies, notable inconsistencies, and implications for curriculum design, learner support, assessment reliability, and residency selection. No formal quality weighting or statistical aggregation was performed.

Effects of Preclinical Pass/Fail Grading

Empirical evidence consistently demonstrates that pass/fail grading in the preclinical years is not associated with inferior performance on standardized licensing examinations. In a national study of U.S. allopathic medical schools, preclinical pass/fail grading was non-inferior to tiered grading with respect to both mean USMLE Step 1 and Step 2 CK scores after adjustment for entering student characteristics [1]. Similarly, single-institution evaluations have shown that although pass/fail grading may be associated with lower performance on internal preclinical examinations, USMLE Step 1 outcomes remain unchanged [2].

Beyond examination performance, preclinical pass/fail grading has been associated with improved learner well-being. Longitudinal cohort studies demonstrate improvements in psychological well-being and learner satisfaction following adoption of pass/fail grading during the first two years of medical school, without adverse effects on Step 1 or Step 2 CK performance, clerkship grades, or residency placement [3]. Collectively, these findings suggest that preclinical pass/fail grading can support learner well-being without compromising downstream standardized examination outcomes [1-3].

Effects of USMLE Step 1 Pass/Fail Reporting

The transition of USMLE Step 1 to pass/fail reporting represents a more consequential shift in the national assessment landscape. Historically, Step 1 functioned as a numerically reported standardized metric with substantial influence on residency selection [4]. Survey-based studies of residency program directors across multiple specialties consistently report anticipated increases in reliance on USMLE Step 2 CK scores, clerkship grades, and narrative evaluations following Step 1 pass/fail implementation [6-8].

Early empirical analyses of applicant outcomes suggest a redistribution of academic signals rather than the elimination of selection pressure. In one early post-transition analysis, applicants with pass/fail Step 1 reporting demonstrated lower mean Step 2 CK scores and fewer traditional markers of academic distinction; however, adjusted analyses revealed no independent association between Step 1 reporting format and match outcomes [9]. These findings underscore the multifactorial nature of residency selection decisions and caution against attributing outcomes to Step 1 reporting changes alone.

Conceptual and empirical analyses further suggest that Step 1 pass/fail reporting may shift, rather than resolve, assessment-related stress. While stress specific to Step 1 may decrease earlier in training, anxiety and assessment-related stress may persist during the dedicated study period and clinical transition, raising concerns that assessment-related pressure may be displaced downstream rather than eliminated [5].

Determinants of USMLE Step 2 Clinical Knowledge Performance

With Step 1 now reported as pass/fail, understanding determinants of Step 2 CK performance has become increasingly important. Step 2 CK assesses applied clinical reasoning and reflects both learner-level academic trajectories and structural features of the clinical curriculum [10-12].

Prior academic performance remains an important predictor of Step 2 CK outcomes. Studies in diverse student populations demonstrate associations between Step 2 CK scores and earlier academic indicators, including Step 1 performance and internal assessments, underscoring continuity in knowledge acquisition and test-taking skills [10]. Clerkship-level assessments also play a critical role. Performance on NBME subject examinations, particularly in internal medicine, in combination with prior academic performance, explains a substantial proportion of variance in Step 2 CK scores [11]. In addition, non-uniform sequencing of core clerkships has been associated with differences in clinical performance and knowledge integration, suggesting cumulative exposure effects during the clinical year [12].

Variability and Equity in Clinical-Year Assessment

Clinical-year assessment systems demonstrate substantial variability across institutions, clerkships, and evaluators. National analyses reveal wide differences in clerkship grading distributions that cannot be fully explained by student performance alone, reflecting institutional norms and assessment culture [13].

Evaluator-related factors further contribute to inconsistency in clinical assessment. Cognitive, social, and environmental sources of bias, including rater subjectivity and contextual influences, have been well-described [14,15]. Analyses of narrative evaluations reveal systematic differences in evaluative language associated with gender and underrepresented-in-medicine status, even among students receiving similar grades, with implications for downstream opportunities [16]. Moreover, small differences in assessed clinical performance may amplify into disproportionately large differences in grades, honors, and awards, particularly affecting students underrepresented in medicine [17].

Although pass/fail reforms were intended in part to mitigate stress and inequity, removal of early numeric metrics may shift evaluative pressure into the clinical environment, where assessment is less standardized and more subjective [3,5]. These dynamics underscore the need for assessment systems that emphasize transparency, reliability, and equity.

Implications for Curriculum Design, Learner Support, and Residency Selection

The reviewed evidence highlights the need to recalibrate curriculum design, learner support, and residency selection practices. Structured transition-to-clerkship courses may support learner adaptation and clarify expectations during the transition to the clinical years [18].

Competency-based and programmatic assessment frameworks emphasize longitudinal assessment, direct observation, and aggregation of multiple low-stakes data points to inform high-stakes decisions [19,20]. In practice, this may include repeated direct observations across clinical settings, structured narrative evaluation prompts aligned with competencies, regular formative feedback, and committee-based review of aggregated performance data. When paired with faculty development and rater calibration, such approaches may enhance the validity and educational impact of clinical-year assessment [19]. However, implementation barriers, including faculty time constraints, assessment literacy, cultural resistance, and data infrastructure, remain substantial. Programmatic assessment should therefore be viewed as a context-sensitive risk-mitigation strategy rather than a prescriptive solution.

Residency selection processes must also adapt to the evolving assessment landscape. Evidence suggests that reliance on multiple complementary assessment signals, rather than a single standardized metric, improves fairness, transparency, and defensibility in selection decisions [21-23]. Interpreting Step 2 CK scores within institutional and curricular context, as part of a holistic review, may mitigate unintended consequences of assessment reform.

Table 1 summarizes key empirical and conceptual studies informing the narrative synthesis.

**Table 1:** Summary of key empirical and conceptual studies included in the narrative review USMLE: United States Medical Licensing Examination; CK: Clinical Knowledge; UCSD: University of California, San Diego; PGME: Postgraduate Medical Education

Author (Year)	Study Type	Population	Focus Area	Key Findings
Kim & George (2018) [1]	National cross-sectional analysis	96 U.S. allopathic medical schools	Preclinical grading systems	Preclinical pass/fail grading was not associated with differences in mean USMLE Step 1 or Step 2 CK scores.
McDuff et al. (2014) [2]	Single-institution retrospective cohort	UCSD medical students	Pass/fail grading	Pass/fail grading lowered internal exam scores but did not affect USMLE Step 1 performance.
Bloodgood et al. (2009) [3]	Single-institution longitudinal cohort	Preclinical medical students	Student well-being	Pass/fail grading improved psychological well-being without adverse effects on USMLE or residency outcomes.
Powell et al. (2022) [7]	National cross-sectional survey	U.S. residency program directors	Residency selection	Program directors anticipate greater reliance on Step 2 CK and holistic review after Step 1 pass/fail.
Lenze et al. (2024) [9]	Retrospective analysis of the STAR survey	2023 U.S. residency applicants	USMLE Step 1 pass/fail	Numeric Step 1 scores were associated with more interview offers, but not with match outcomes after adjustment.
Fitz et al. (2022) [11]	Multi-institutional retrospective cohort	U.S. medical students (30+ schools)	Step 2 CK predictors	NBME subject exams and clerkship characteristics explained substantial variance in Step 2 CK scores.
Alexander et al. (2012) [13]	National cross-sectional survey	U.S. clerkships	Grading variability	Marked variability in clerkship grading systems limits comparability across institutions.
Zaidi et al. (2018) [15]	Generalizability analysis	Core clerkship students	Assessment reliability	Clinical competency assessments showed low reliability and high assessor-dependent variance.
Rojek et al. (2019) [16]	Linguistic analysis	Clerkship narrative evaluations	Assessment bias	Evaluative language differed by gender and URM status despite similar grades.
Teherani et al. (2018) [17]	Single-institution cohort study	Medical students	Equity in assessment	Small differences in assessment are amplified into large disparities in honors and AOA selection.
Hauer et al. (2014) [19]	Narrative review	—	Entrustment decisions	Identified trainee, supervisor, task, and context factors influencing entrustment.
ten Cate & Scheele (2007) [20]	Conceptual analysis	—	Competency-based education	Introduced entrustable professional activities (EPAs) to link competencies with clinical practice.
Schuwirth & van der Vleuten (2011) [21]	Conceptual framework	—	Programmatic assessment	Advocates longitudinal aggregation of low-stakes assessments to support valid high-stakes decisions.
Patterson et al. (2016) [22]	Systematic review	UGME and PGME selection studies	Selection methods	Single academic metrics incompletely predict long-term outcomes; multi-signal approaches improve fairness.

Limitations

This review has several limitations inherent to its narrative design. The literature was not synthesized using systematic review methodology, and a formal risk-of-bias assessment was not performed. Although efforts were made to prioritize national and multi-institutional studies, selection bias remains possible. The reviewed literature is predominantly drawn from U.S. allopathic medical education contexts, which may limit generalizability to other training systems. In addition, many studies examining the consequences of USMLE Step 1 pass/fail reporting reflect early post-transition cohorts, and longer-term effects on learner outcomes and residency selection remain incompletely characterized.

## Conclusions

Validated evidence demonstrates that pass/fail preclinical grading is associated with improved learner well-being and does not predict inferior performance on standardized licensing examinations. In contrast, USMLE Step 1 pass/fail reporting appears to redistribute evaluative emphasis toward USMLE Step 2 CK and clinical-year assessment, potentially shifting assessment-related pressure into less standardized environments. Step 2 CK performance reflects both learner-level academic trajectories and features of the clinical curriculum, underscoring the importance of equitable, clinically meaningful assessment systems. Medical schools should prioritize transparent, competency-based assessment approaches during the clinical years, while residency programs should interpret Step 2 CK within an institutional context as part of holistic applicant evaluation.
